# Factors Influencing the Accessibility and Reliability of Health Information in the Face of the COVID-19 Outbreak—A Study in Rural China

**DOI:** 10.3389/fpubh.2021.706779

**Published:** 2021-12-23

**Authors:** Li Zhu, Zixuan Peng, Shaohui Li

**Affiliations:** ^1^School of Politics and Public Administration, Guangxi University for Nationalities, Nanning, China; ^2^Institute of Health Policy, Management and Evaluation, Dalla Lana School of Public Health, University of Toronto, Toronto, ON, Canada; ^3^College of Informatics, HuaZhong Agricultural University, Wuhan, China

**Keywords:** accessibility of health information, reliability of health information, COVID-19, information sources, information channels, rural China

## Abstract

**Introduction:** Rural residents have been shown to have limited access to reliable health information and therefore may be at higher risks for the adverse health effects of the COVID-19. The aim of this research is 2-fold: (1) to explore the impacts of demographic factors on the accessibility of health information; and (2) to assess the impacts of information channels on the reliability of health information accessed by rural residents in China during the COVID-19 outbreak.

**Methods:** Mixed methods research was performed to provide a relatively complete picture about the accessibility and reliability of health information in rural China in the face of the COVID-19. A quantitative research was conducted through surveying 435 Chinese rural residents and a qualitative study was performed through collecting materials from one of the most popular social media application (WeChat) in China. The logistic regression techniques were used to examine the impacts of demographic factors on the accessibility of health information. The Content analysis was performed to describe and summarize qualitative materials to inform the impacts of information channels on the reliability of health information.

**Results:** Age was found to positively associate with the accessibility of health information, while an opposite association was found between education and the accessibility of health information. Rural residents with monthly income between 3,001 CNY and 4,000 CNY were the least likely to access health information. Rural residents who worked/studied from home were more likely to access health information. Meanwhile, health information tended to be derived from non-official social media channels where rumors and unverified health information spread fast, and the elderly and less-educated rural residents were more likely to access health misinformation.

**Conclusions:** Policy makers are suggested to adopt efficient measures to contain the spread of rumors and unverified health information on non-official social media platforms during the outbreak of a pandemic. More efforts should be devoted to assist the elderly and less-educated rural residents to access reliable health information in the face of a pandemic outbreak.

## Introduction

The Wuhan Municipal Health Commission declared the outbreak of a newly discovered coronavirus disease (COVID-19) at the end of 2019 (1), during which time a large amount of migrant workers returned to their home in villages to celebrate the Chinese Lunar New Year without realizing that the COVID-19 had spread fast and widely among them, bringing substantial risks to people living in rural areas where health services are less accessible and of poor quality (2, 3). During a pandemic, quality information is essential to keep the rural residents calm and informed on the correct steps to take (4). Traditionally, media such as television and newspapers, which carry information from authorized sources, played a central role in transmitting reliable health information (5). In recent years, the internet has become an increasingly popular source of health information (6). Despite the importance of social media as a platform for individuals to access free health information has been widely recognized, the unregulated nature of the internet can result in the spread of invalidated or unproven information (6). Health misinformation about the COVID-19 is a serious threat to global public health and can lead to life-threatening consequences (7); therefore, it is of paramount significance to investigate whether rural residents can access reliable health information during the outbreak of the COVID-19.

Although the demographic characteristics of media users have been described by a previous research (8), no study has yet assessed how specifically these factors would impact the accessibility of health information during a pandemic in rural China. Moreover, the media landscape has dramatically changed over the past decade, with traditional media (e.g., newspapers and television) now supplemented and overlapped by social media (e.g., blogs and discussion forums) (9). Various Social media applications, such as QQ and WeChat, have been widely used by rural residents in China as a result of improved affordability of mobile technology (10). Furthermore, previous research has demonstrated that the prevalence of social media offers unprecedented opportunities to enable under-served population to access health information. For example, social media was demonstrated to be an important avenue to access information on prevention, control and treatment of Ebola epidemics (11). However, the use of social media has been shown to accelerate the dissemination of inaccurate health information (12–14), which in turn may trigger public panic and even result in detrimental adverse consequences (15). Additionally, as social media has been widely accepted as an important platform for public health information by government officials, public health agencies, and the general population (16), multiple channels of social media has been brought into focus, and the media credibility discrepancy between official channels and non-officail channels including interpersonal communication channel and we-media channel have been documented (17). Nevertheless, it remains unclear about the reliability of health information accessed from different information conduits. For these considerations, a comprehensive investigation on the accessibility and reliability of health information accessed via either official or non-official social media channels helps to provide valuable implications for rural residents facing the pandemic.

This study aims to address the following two questions: first, to investigate the impacts of demographic factors on the accessibility of health information during the outbreak of the COVID-19; and second, to evaluate the impacts of information channels on the reliability of health information accessed. This paper will be structured in the following manner: the next section will introduce our study design, data sources, variables, and data analysis methods. Key research findings will be outlined in Section Results before discussed in Section Discussion. We ended with providing research conclusions and possible policy implications.

## Methods

### Study Settings

Previous research demonstrated that the top five channels for the public to obtain information during the outbreak of the Severe Acute Respiratory Syndrome (SARS) were TV (85.4%), interpersonal communication (53.4%), newspaper (48.5%), radio (39.6%), and conference communication (22.6%) (18). But now the study settings have changed considerably, as the top three channels for obtaining information on the COVID-19 prevention and control were press release (91.50%), internet (74.05%), WeChat, QQ and other communication softwares (66.22%) (19). As such, our study focused on the accessibility and reliability of health information in the face of the COVID-19 outbreak in the rural community setting in China.

### Study Design

To address the complex health problem proposed by our research, we performed a mixed methods research: first, we started with conducting a quantitative study through surveying Chinese rural residents to reveal the impacts of demographic factors on accessing health information; and subsequently, a qualitative study was carried out through collecting data from a social media platform to inform the impacts of information channels on the reliability of health information accessed.

### Data Sources

Quantitative data were collected from February 14 to February 22, 2020 using a self-designed questionnaire consisting information on the prevention and control measures, the demographic characteristics of the research participants, and their accessibility of health information during the outbreak of the COVID-19. We distributed questionnaires online and finally obtained feedback from 435 rural residents in 28 provinces in China. All of the questionnaires were valid after checking for the missing and invalid values.

The qualitative data were collected from January 21 to February 7, 2020. Qualitative data were collected from WeChat considering that it is one of the most popular social media application in rural China (10) and the ease of tracking information sources on it. Specifically, we collected chats from the WeChat chat rooms and posts from the WeChat “moments.” Chats and posts that have more pertinent content were chosen purposefully and we stopped collecting materials when the theory was detailed sufficiently or when the categories were saturated (20). Finally, qualitative materials were collected from 5 WeChat chat groups and 15 WeChat “moments.”

### Variables

The accessibility of health information was the outcome of our interest. The accessibility of health information was measured by an ordered categorical variable using a 5-point Likert scale (1 = not at all, 2 = only a little, 3 = to some extent, 4 = rather much, or 5 = very much). Age (years), monthly income (CNY), education (postgraduate education and above, undergraduate education, secondary education, or primary education and below), political identity (the China Communist Party, the China Communist Youth League, the Democratic party, or masses), personal role (government officials, medical staff, ordinary residents, or people returned to visit relatives), and employment (returned to work/study, waited to return to work/study, work/study from home, retired at home, or unemployed) were demonstrated by previous studies to impact the accessibility of health information (21–24), and therefore were included as our independent variables.

The reliability of health information was measured by whether the participant acknowledged reliable health information or refuted unreliable health information during the outbreak of the COVID-19. The investigators focused on the association between information channels and the reliability of health information. Specifically, we classified social media into official and non-official social media channels to explore how they associate with the reliability of health information. Additionally, our research also investigated the impacts of age and education on the association between information channels and the reliability of health information (25).

### Data Analysis Methods

Since our dependent variable is ordered, we constructed an ordered logit regression model to examine the impacts of demographic factors on the accessibility of health information. The Omnibus Tests of Model Coefficients were reported to examine whether all the coefficients were jointly significant. The Hosmer and Lemeshow test was performed to examine the goodness-of-fit of the regression model. All the quantitative analyses were conducted using the R statistical software (26).

The Content analysis method was used to analyze qualitative materials. We started with compiling qualitative materials into a single text consisting of 6,209 Chinese characters. Subsequently, we performed selective coding on qualitative materials to identify key concepts or themes according to our research hypotheses. Finally, we counted the occurrence of these different concepts or themes, and analyzed how age and education impact the association between information channels and the reliability of information. The qualitative analyses were conducted using the Nvivo 11 software.

## Results

### Quantitative Results

#### Descriptive Results

Table 1 reports descriptive statistics for our research sample. There were 435 research participants in our study, of which 39.08% (*n* = 170) were from Hubei province. Specifically, 86 (*p* = 19.77%) and 83 (*p* = 19.08%) research participants were in Wuhan and Huanggang city, respectively. The majority of our research participants aged between 19 and 30 (*n* = 190; *p* = 43.68%), had secondary education (*n* = 146; *p* = 33.56%), waited to return to work/study (*n* = 151; *p* = 34.71%), were ordinary residents (*n* = 355, 81.61%), had monthly income <3,001 CNY (*n* = 193, 44.37%), did not have political identity (*n* = 214, 49.20%). The number of family member averaged at 7.56 with a SD of 1.63.

**Table 1:** Descriptive statistics for the research sample.

**Variables**	**Categories**	**Number (percentage)**
Age	≤ 18	20 (4.60%)
	(19-30)	190 (43.68%)
	(31-40)	158 (36.32%)
	(41-50)	52 (36.32%)
	(51-60)	12 (2.76%)
	>60	3 (0.69%)
Education	Postgraduate education and above	144 (33.10%)
	Undergraduate education	68 (15.63%)
	Secondary education	146 (33.56%)
	Primary education and below	77 (17.70%)
Employment	Study/work in universities/companies	103 (23.68%)
	Waited to return to work/study	151 (34.71%)
	Study/work from home	95 (21.84%)
	Retired at home	4 (0.92%)
	Unemployed	82 (18.85%)
Income	≤ 1,205 CNY	109 (25.06%)
	(1,206-2,000 CNY)	40 (9.20%)
	(2,001-3,000 CNY)	44 (10.11%)
	(3,001-4,000 CNY)	67 (15.40%)
	(4,001-5,000 CNY)	60 (13.79%)
	> 5,000 CNY	115 (26.44%)
Political identity	The China Communist Party	105 (24.14%)
	The China Communist Youth League	109 (25.06%)
	The Democratic party	7 (1.61%)
	Masses	214 (49.20%)
Personal role	Government officials	17 (3.91%)
	Medical staff	4 (0.92%)
	Ordinary residents	355 (81.61%)
	People returned to visit relatives	59 (13.56%)
Family members	Number of family members [mean (SD)]	7.56 (1.632)
Accessibility of	Not at all	11 (2.53%)
health information	Only a little	19 (4.37%)
	To some extent	228 (52.41%)
	Rather much	126 (28.97%)
	Very much	51 (11.72%)

*The total number of research participants were 435*.

About 50 research participants (*p* = 11.49%) reported there were confirmed cases of the COVID-19 in their villages. About 81.15% of the research participants (*n* = 353) had been stranded in Wuhan for more than 2 weeks since the outbreak of the COVID-19. Besides, more than half of the research participants (*n* = 283) were satisfied with the village committees about their work on preventing and controlling the pandemic, with 197 (*p* = 45.29%) reporting to be very satisfied and 86 (*p* = 19.77%) reporting to be moderately satisfied.

#### Factors Influencing the Accessibility of Health Information

Table 2 reports the regression results about the impacts of demographic factors on the accessibility of health information. The odds of having access to health information were found to be more than 9 times higher (OR = 9.24; 95% CI: 1.33 and 64.25) among participants aged between 51 and 60 than participants aged ≤ 18. Besides, participants with primary education and below (OR = 4.10; 95% CI: 1.59 and 10.60) and with secondary education (OR = 3.30; 95% CI: 1.44 and 7.57) were more likely to access health information than participants with postgraduate education and above. Additionally, compared with people whose monthly income was <3,001 CNY or more than 4,000 CNY, people with monthly income between 3,001 CNY and 4,000 CNY were the least likely to access health information (OR = 0.33; 95% CI: 0.148 and 0.722). Furthermore, rural residents who worked/studied from home were more likely to access health information compared to those who had returned to work/study, respectively. The *p*-value of the Omnibus Tests was <0.05, rejecting the null hypothesis that all the regression coefficients were 0. The *p*-value of the Hosmer and Lemeshow test was larger than 0.05, implying that the regression model was well-fitted.

**Table 2:** The impacts of demographic factors on the accessibility of health information.

**Demographic factors**	**Estimate**	**OR**	**Standard error**
**Age**			
≤ 18	Ref	Ref	Ref
(19–30)	−0.340	0.712	0.637
(31–40)	−0.117	0.890	0.603
(41–50)	0.963	2.619	0.636
(51–60)	2.224**	9.244	0.989
>60	22.718	7351048873.000	40192.977
**Education**			
Postgraduate education and above	Ref	Ref	Ref
Undergraduate education	0.337	1.401	0.379
Secondary education	1.193***	3.297	0.424
Primary education and below	1.411***	4.100	0.484
**Political identity**			
The China communist party	Ref	Ref	Ref
The China communist youth league	−0.637*	0.529	0.346
The democratic party	−0.699	0.497	0.864
Masses	−0.465	0.628	0.346
**Personal role**			
Government officials	Ref	Ref	Ref
Medical staff	−0.930	0.394	1.403
Ordinary residents	−0.202	0.817	0.632
People returned to visit relatives	0.226	1.253	0.682
**Employment**			
Work/study in universities/companies	Ref	Ref	Ref
Waited to return to work/study	0.363	1.438	0.314
Work/study from home	0.639*	1.894	0.336
Retired at home	−22.263	0.000	40192.977
Unemployed	−0.344	0.709	0.436
**Income**			
≤ 1,205 CNY	Ref	Ref	Ref
(1,206-2,000 CNY)	−0.145	0.865	0.436
(2,001-3,000 CNY)	−0.827*	0.437	0.453
(3,001-4,000 CNY)	−1.116***	0.327	0.404
(4,001-5,000 CNY)	−0.925**	0.397	0.403
> 5,000 CNY	−0.695**	0.499	0.337
**Number of family member**	−0.038	0.963	0.064
The omnibus tests of model coefficients	<0.001 (*df* = 24, Chi-square = 78.734)		
The Hosmer and Lemeshow test	0.783 (*df* = 8, Chi-square = 4.755)		

* *p < 0.10*;

** *p < 0.05*;

*** *p < 0.01*.

Table 3 reports the utilization of different information sources during the COVID-19 outbreak. About a third of our research participants obtained health information using traditional media, such as television, radio, and newspaper. Although traditional media remained to be an important information source, new media had received increased popularity that more than 40% of our research participants used various types of social media, such as WeChat, QQ, WeBlog, Tiktok, and Toutiao, to acquire health information related to the COVID-19.

**Table 3:** Utilization of different information sources.

**Mean (percentage)**	**Wearing masks**	**Washing hands**	**Keeping rooms ventilated**	**Other health information**
Traditional media	303 (33.592%)	303 (34.122%)	295 (33.676%)	292 (37.922%)
New media	379 (42.018%)	382 (43.018%)	385 (43.950%)	391 (50.779%)
Acquaintances	163 (18.071%)	159 (17.905%)	153 (17.466%)	43 (5.584%)
Other sources	57 (6.319%)	44 (4.955%)	43 (4.909%)	44 (5.714%)
Sum	902	888	876	770

### Qualitative Results

#### Diachronic Description

At the very beginning of the outbreak of the COVID-19, only the young and well-educated were engaging in discussing, forwarding, and posting health information about the COVID-19 in the WeChat chat rooms and “moments.” It was until the Wuhan health department declared the number of confirmed cases of the COVID-19 on January 3, 2020 and the health professionals emphasized the severity of the coronavirus, health information about the COVID-19 went viral in the WeChat chat rooms and “moments.”

#### Encoding Matrix Analyses

Table 4 describes the nodes of qualitative materials. Three forms of notes were recorded by researchers: first, channels through which rural residents accessed health information related to the COVID-19; second, reliability of health information accessed by these rural residents; and third, age and education of these rural residents who discussed, forwarded, and posted health information about the COVID-19. Table 5 reports the impacts of information channels on the reliability of health information. Information related to the COVID-19 tended to be derived from non-official social media channels where rumors and unverified health information were frequently reported. The young and well-educated people were less likely to access health misinformation, which is corroborated with the finding that the young and well-educated people were more likely to access reliable health information.

**Table 4:** Nodes of qualitative description.

**Tree nodes**	**Free nodes**		**Qualitative materials**
Accessibility of health information	Information sources	Official channels	This health information was forwarded from official WeChat accounts, such as the People's Daily^a^...
		Non-official channels	This health Information was published by non-official agencies and individuals on “TikTok”...
Reliability of health information accessed	Reliable health information	Accepting reliable Information about COVID-19	I obtain health information related to wearing masks, washing hands, and keeping rooms ventilated from “Toutiao”^b^... The guidance on preventing COVID-19 issued by the government departments could be seen from official WeChat accounts... I see some articles and comments about the prevention and treatment of COVID-19 on People's Daily...
		Rejecting unreliable health information	I think health information provided by that video is incorrect…
	Unreliable health information	Accepting unverified health information	I think this virus can only survive for short period of time when the temperature is above 20 degree and thus, heating equipment should be used and windows should be closed…
		Accepting rumors	I agree that eating eggs could prevent the virus…
Demographic factors	Age	Old people	This was forwarded from an old people... The author's aunt made and published the video...
		Young people	People who sent me the health information is very young...
	Education	Well-educated people	Health information about COVID-19 was forwarded from an educated person...
		Less-educated people	People who spread the rumors that eating eggs could prevent the virus was not well-educated...

a *The People's Daily is the largest newspaper group in China*.

b *“Toutiao” (Jinri Toutiao) is a Chinese news and information content platform*.

**Table 5:** The impact of information channels on the reliability of health information.

	**Social media**		**Age**		**Education**	
	**Non-official channels**	**Official channels**	**Old people**	**Young people**	**Less-educated people**	**Well-educated people**
**Reliable health information (frequency)**						
Accepting reliable health information	8	11	3	10	2	10
Rejecting unreliable health information	3	1	0	3	1	1
**Unreliable health information (frequency)**						
Accepting unverified information	3	0	3	0	3	0
Accepting rumors	12	0	12	0	12	0

## Discussion

Our study aims to explore whether rural residents could access reliable health information during the COVID-19 outbreak. Our research demonstrated that in rural China, the elderly, the less-educated, and residents who worked/studied from home were more likely to access health information. Rural residents with monthly income between 3,001 CNY and 4,000 CNY were the least likely to access health information. Meanwhile, health information tended to be derived from non-official social media channels where rumors and unverified health information were frequently present, and these misinformation were more likely to be accessed by the elderly and less-educated rural residents.

Quantitative research demonstrated that the associations between demographic factors and the accessibility of health information varied in the context of the COVID-19. In line with prior research (27), access to employment were positively associated with the accessibility of health information. Rural residents aged between 51 and 60 were less likely to have difficulty accessing health information, which is consistent with previous research that reported older people constitute a growing group of health information seekers on the internet (24, 28). Nevertheless, an unanticipated finding was that rural residents with lower education levels were more likely to obtain health information, which is at variance with a previous research (29). Similarly, in contrast to a previous study (28), our study demonstrated that compared with rural residents whose monthly income were <3,001 CNY or more than 4,000 CNY, those with monthly income between 3,001 CNY and 4,000 CNY were the least likely to access health information (30). The findings may be explained by the rapid establishment and development of information infrastructure in rural China, which makes it possible for the old, less-educated, and poor rural residents to access health information using social media during the outbreak of COVID-19. Another possible explanation is that compared with the younger and well-educated group, the elderly and less-educated group tend to be passive information-takers.

Qualitative study further demonstrated that health information related to the COVID-19 tended to be derived from non-official social media channels where rumors and unverified health information were always found. At the early stage of the pandemic, reliable information about the prevention, control, and treatment of the COVID-19 were spread via official social media channels, which is consistent with a previous Chinese study focusing on the Dengue Epidemic in Guangzhou (31). But when health information about the COVID-19 went viral, official social media channels were unable to provide sufficient health information in a timely manner, which led to the explosion of non-official social media. Our study also provided preliminary evidence that the elderly and less-educated groups were less likely to access reliable health information compared with the young and well-educated rural residents in the face of a pandemic. This may be explained by the fact that increased access to health information increases the probability of accessing health misinformation. Another possible explanation is that young and well-educated people were more skeptical about health information present on social media, and they tended to consider social media as a channel for networking and entertainment rather than a resource for health information (12, 13).

Our research focused on Chinese rural residents, a marginalized but more vulnerable population group, contributing to advance knowledge in the field of health communication in the face of a pandemic. There were 509.79 million rural residents in China in 2020, which accounts for 36.11% of the total population (32). About 23.8% of the rural population in 2020 were older persons, while the percentage of the urban population was smaller at 15.5% (32). More than 98% of the household owners in rural China had secondary education and below in 2019 (33). These facts highlights the importance of our research in terms of shedding light on how to help these vulnerable population groups in the face of a pandemic. Besides, our research distributed questionnaires in the early days of the outbreak and retrieved qualitative materials from a social media application, having the strength in reducing possible recall bias. Moreover, our findings can be representative of the situation in China given that our quantitative research had a large sample size consisted of rural residents from different provinces. However, some limitations warranted recognition. First, confronted with the isolation measure of the COVID-19, we surveyed our research participants online, which may impair the validity of our research findings. Second, our qualitative study only considered social media channels, which may be insufficient to provide a complete picture of the association between the reliability and sources of health information. Further research is suggested to compare how various information sources are associated with the reliability of information. Finally, our study use a subjective measure to assess the reliability of health information, while whether consistent findings can be obtained for studies with objective measures deserves further exploration.

## Conclusion

Our research explored the impacts of demographic factors on the accessibility of health information, and how social media channels impact the reliability of health information in the face of the COVID-19. Our research found that in rural China, the elderly, the less-educated, and residents who worked/studied from home were more likely to access health information. Besides, compared with rural residents whose monthly income were <3,001 CNY or more than 4,000 CNY, those with income between 3,001 CNY and 4,000 CNY were the least likely to access health information. Moreover, health information related to the COVID-19 tended to be derived from non-official social media channels, and the elderly and less-educated rural residents were more susceptible to unreliable health information. These findings may inspire policymakers to propose appropriate policy measures to help the most vulnerable group to access reliable health information in the face of a pandemic. Our findings also imply that we should be cautious about the role of social media in spreading reliable health information during the early days of the pandemic outbreak.

## Data Availability Statement

The raw data supporting the conclusions of this article will be made available by the authors, without undue reservation. The datasets used and/or analysed during the current study are available from the corresponding author on reasonable request.

## Ethics Statement

Ethical review and approval was not required for the study on human participants in accordance with the local legislation and institutional requirements. Written informed consent to participate in this study was provided by the participants' legal guardian/next of kin. We requested and obtained the permission from our participants to do scientific research by using the data collected, and we confirmed that all the data were restricted to scientific research and all the information were anonymous

## Author Contributions

LZ and ZP drafted the manuscript and contributed to collecting research materials and performing data analyses. LZ, ZP, and SL revised the manuscript. All authors contributed to the article and approved the submitted version.

## Funding

The design of the study, the collection, analysis, and interpretation of data, and the writing of the manuscript were supported by the Project of Guangxi Philosophy and Social Science—Study on the Resilience of Health Governance System in Guangxi region under public health emergencies (20CGL003).

## Conflict of Interest

The authors declare that the research was conducted in the absence of any commercial or financial relationships that could be construed as a potential conflict of interest.

## Publisher's Note

All claims expressed in this article are solely those of the authors and do not necessarily represent those of their affiliated organizations, or those of the publisher, the editors and the reviewers. Any product that may be evaluated in this article, or claim that may be made by its manufacturer, is not guaranteed or endorsed by the publisher.
